# Supplementary material for: Outer Membrane Vesicles of Avian Pathogenic Escherichia coli Mediate the Horizontal Transmission of blaCTX-M-55
Source: Pathogens. 2022 Apr 18;11(4):481. doi: 10.3390/pathogens11040481 (PMC9025603; doi:10.3390/pathogens11040481)
Supplement: Supplementary file 1 [file pathogens-11-00481-s001.zip › Table S1.pdf]

Table S1 Minimal inhibitory concentrations, gene and plasmid analysis of the bacteria used in this study

Strain	Antibiotic (μg/mL)									Resistance Gene	Plasmid
	CAZ	CTX	AML	AMP	MEM	ATM	FOX	ENR	FFC		
SCAO22	512	512	512	512	<0.5	256	32	16	128	*bla*$_{CTX-M-55}$, *bla*$_{TEM-1B}$, *aac(3)-IV*, *aph(3')-Ia*, *sul2*, *tet(B)*, *catA1, floR, dfrA17*, *aph(6)-Id*, *aph(6)-Id*, *mph(A)*, *sitABCD*	IncI2, Col440I, IncFIB, p0111, IncFIC, IncI, IncQ1
EC600	1	<0.5	8	8	<0.5	<0.5	2	<0.5	<0.5	ND	ND
OMV-EC600	512	512	512	512	<0.5	256	32	8	64	*bla*$_{CTX-M-55}$*, bla*$_{TEM-1B}$, *floR*, *aac(3)-IV*	IncI2, IncI, Col440I
AMLOMV-EC600	512	512	512	512	<0.5	256	32	8	64	*bla*$_{CTX-M-55}$*, bla*$_{TEM-1B}$, *floR*, *aac(3)-IV*	IncI2, IncI, Col440I
ENROMV-EC600	512	512	512	512	<0.5	256	32	8	64	*bla*$_{CTX-M-55}$*, bla*$_{TEM-1B}$, *floR*, *aac(3)-IV*	IncI2, IncI, Col440I

ND: not detected; the MIC resistance breakpoints of Cefotaxime (CTX), Ceftazidime (CAZ), Amoxicillin (AML), Ampicillin (AMP), Meropenem (MEM), Aztreonam (ATM), Cefoxitin (FOX), Florfenicol (FFC) are 2 μg/mL, 4 μg/mL, 8 μg/mL, 8 μg/mL, 8 μg/mL, 4 μg/mL, 8 μg/mL, and 8 μg/mL .
