# Supplementary material for: Outer Membrane Vesicles of Avian Pathogenic Escherichia coli Mediate the Horizontal Transmission of blaCTX-M-55
Source: Pathogens. 2022 Apr 18;11(4):481. doi: 10.3390/pathogens11040481 (PMC9025603; doi:10.3390/pathogens11040481)
Supplement: Supplementary file 1 [file pathogens-11-00481-s001.zip › Table S2.pdf]

Table S2 Physical characteristics of OMVs isolated from *E. coli* SCAO22

Treatment	Antibiotic concentration (μg/mL)	Mean size distribution [a] （nm）	Concentration [b] （Particles/mL）	Copies of $bla_{CTX-M-55}$ (copies/μL)	Transfer frequency (CFU/mL)
No treatment	-	79.42	$2.26\pm0.78x10^{10}$	208	$4.17\pm2.13x10^{-6}$
AML treatment	128	60.14	$8.89\pm0.36x10^{11}$	1196	$4.26\pm2.47x10^{-6}$
ENR treatment	4	64.18	$5.66\pm1.2x10^{12}$	30615	$6.8\pm2.14x10^{-5}$

a: The average of the three experiments

b: The volume of the medium used for OMV extraction was 400 mL
